# Ascorbate Peroxidase from *Leishmania major* Controls the Virulence of Infective Stage of Promastigotes by Regulating Oxidative Stress

**DOI:** 10.1371/journal.pone.0011271

**Published:** 2010-06-23

**Authors:** Swati Pal, Subhankar Dolai, Rajesh K. Yadav, Subrata Adak

**Affiliations:** Division of Structural Biology & Bio-Informatics, Indian Institute of Chemical Biology, Kolkata, West Bengal, India; Federal University of São Paulo, Brazil

## Abstract

**Background:**

Peroxidase represents a heterogeneous group of distinct enzyme family that plays extremely diverse biological functions. Ascorbate peroxidase from *Leishmania major* (LmAPX) has been shown to be central to the redox defense system of *Leishmania*. To investigate further its exact physiological role in *Leishmania*, we attempted to create LmAPX -knockout mutants by gene replacement in *L. major* strains.

**Methodology/Principal Findings:**

The null mutant cell culture contains a higher percentage of metacyclic and apoptotic cells compared to both wild type and LmAPX overexpressing cells. Flowcytometric analysis reveals the presence of a higher concentration of intracellular H_2_O_2_, indicative of increased oxidative stress in parasites lacking LmAPX. IC_50_ value for exogenously added H_2_O_2_ shows that deletion of LmAPX in *L. major* renders the cell more susceptible to H_2_O_2_. Real time PCR studies demonstrate an elevated mRNA level of non-selenium glutathione peroxidase in LmAPX null mutant cell line, suggesting that these enzymes were induced to compensate the LmAPX enzyme. The null mutant cells exhibit hypervirulence after infection with macrophages as well as inoculation into BALB/c mice; in contrast, overexpressing cells show avirulence.

**Conclusions/Significance:**

Collectively, these data provide strong evidence that LmAPX is an important factor for controlling parasite differentiation and survival within macrophages.

## Introduction

*Leishmania* are protozoan human pathogens that cycle between an extracellular promastigote stage residing in the digestive tract of vector sandflies and an intracellular amastigote stage colonizing the phagolysosomal compartment of mammalian macrophages. By nature, the transformation of replicating, poorly infective procyclic promastigote into non-replicating, highly infective metacyclic promastigote occurs within the insect vector [1]. The transformation of procyclic to metacyclic form is accompanied by an improved ability to infect and survive in the vertebrate host, where the parasite is assaulted by the host's immune system [2]. During the differentiation from procyclic to metacyclic forms in the insect gut, morphological and chemical changes like shorter cell size, slender shape, longer flagellum, elevated level of both lipophosphoglycan (LPG) and surface protease gp63 expression are observed [3], [4]. When cultivated in liquid medium, *L. major* promastigotes transform from a less infective procyclic form during log-phase growth to a highly infectious metacyclic promastigote when they reach stationary phase [5], [6]. This development is accompanied by an increased ability to survive intracellularly in macrophages. Although several metacyclic stage specific genes have been identified yet the molecular mechanism(s) for the initiation and regulation of these genes remain uncertain.

It is a well-known phenomenon that phagocytic functions are suppressed by the apoptotic cells (AnxA5^+^) via an immune-silencing (phagocytotic) process [7], [8]. The immune-silencing phagocytotic process is characterized by the release of antiinflammatory cytokines such as transforming growth factor beta (TGF-β) and IL-10, and down-regulation of the proinflammatory cytokine tumor necrosis factor alpha (TNF-α.). Recently a group of workers have shown that neither 100% nonapoptotic metacyclic (AnxA5^-^) nor 100% apoptotic metacyclic *L. major* promastigote (AnxA5^+^) population leads to the establishment of disease; rather a mixture of nonapoptotic and apoptotic parasites goes ahead to the development of severe disease [9], [10]. This *in vivo* study suggests that virulence is found to depend on the presence of apoptotic parasites that is crucial for both disease development and intracellular survival of the parasite as a population.

Much progress has been made in recent years to understand the signaling function of exogenously added reactive oxygen species (ROS) (e.g. stimulation with hydrogen peroxide), NADPH oxidase-generated ROS or mitochondria-generated ROS [11], [12]. Some signaling molecules and pathways have been already identified that are explicitly activated by increases in mitochondrial ROS. For example, the c-Jun N-terminal kinase (JNK) is activated by mitochondria-generated ROS and induces apoptosis through the regulation of cytochrome c release and caspase activation [13]. Both proapoptotic signaling molecules, JNK and p38, are activated by high doses of ROS [14]. Cells also have protective ROS-sensing signaling pathways that are activated by moderate increases in mitochondrial ROS [12]. Although several roles of mitochondrial-generated ROS in multicellular organism have been identified, almost nothing is known about the molecular function(s) of mitochondria-generated ROS in *Leishmania*. Recently, we have demonstrated that overexpression of LmAPX in *L. major* protects promastigote cells against oxidative stress-induced apoptosis [15]–[17]. Peroxidase is found in a great variety of organisms, where they fulfill distinct functions, such as ROS detoxification, cell signaling, or differentiation [18]. LmAPX, localized in the mitochondrion, seems to be of particular interest, since kinetoplastids are known to generate H_2_O_2_ as a by-product of their own mitochondrial energy metabolism. Although localization and overexpression studies have predicted the possible physiological role of this protein, the exact physiological function is unknown. In this manuscript, we provide evidence for the first time that LmAPX gene knockout cells have higher levels of intracellular H_2_O_2_ and elevated level of both metacyclic and apoptotic cells, more infectious property with respect to macrophage infection, and induced larger cutaneous lesions (hypervirulence). Taken together, these gene knockout results not only support the central role of ROS in the mechanism of *Leishmania* differentiation, but also indicate its relevance to the control of virulence during evolution.

## Results

### Generation of LmAPX Null Mutants

**Figure 1:**
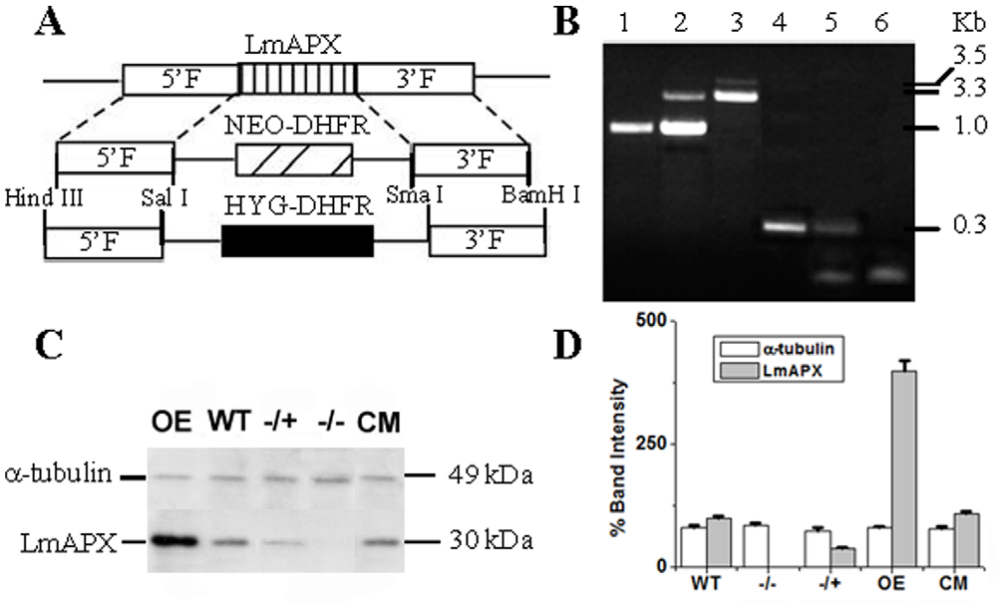
Targeted gene replacement of LmAPX alleles. (A) Schematic representation of the LmAPX locus and the plasmid constructs used for gene replacement. (B) Agarose gel analysis of PCR amplified products of LmAPX gene. Lanes 1/4, 2/5 and 3/6 correspond to PCR with gDNA from WT, APX/APX::NEO heterozygous mutants and KO mutants respectively with primers external (lanes 1-3) and internal (lanes 4-6) of LmAPX gene. External forward and reverse primers were generated from 28 basepair upstream of the LmAPX gene and position 34 downstream of the stop codon of gene, respectively. The expected size of the LmAPX, NEO and HYG gene PCR product are 1.0, 3.3 and 3.5 Kb, respectively. Internal forward and reverse primers were generated from positions 360 and 623 basepair of the gene, respectively. The expected size of the PCR product from WT and APX/APX::NEO heterozygous mutants is 0.263 Kb (C) Western blot results using anti-APX and anti-α-tubulin antibody. 200 µg of *L. major* lysate were used for Western blotting. All the data are representative of at least three independent experiments. (D) Bar diagram depicting the % band intensities in the Western blot. Band intensity was quantified by Total Lab TL100 software (Nonlinear Dynamics Ltd) and error bars represent the SD from three independent experiments. Band intensity of wild type LmAPX was considered as a 100%. The graphs were assembled by using Origin 7.0 software (Microcal Software, Inc. Northampton, MA).

*L. major* Gene DB reveals that LmAPX is a single copy gene. To find out biological significance of this protein in the parasite we attempted to create LmAPX null mutant cell line by gene replacement. *Leishmania* are asexual diploids and thus require two rounds of gene replacement [19]. We generated LmAPX knockout constructs by gene replacement either hygromycin or neomycin selectable markers (Fig. 1A). After two rounds of transfection and selection with neomycin and hygromycin drugs, both the LmAPX alleles in cells had been replaced. To screen the null mutants, we first performed a PCR analysis on genomic DNA with primers generated from the coding region as well as from the 5′- and 3′- flanking regions (Fig. 1B). Western blot analysis with anti-LmAPX antibody further confirmed the absence of LmAPX in the LmAPX ^−/−^ line, and the presence of ∼40% LmAPX in the LmAPX^−/+^ line (Fig. 1C–D). These results suggested that both LmAPX alleles had been knocked out in the *L. major* cell line that is resistant to both neomycin and hygromycin drugs. On the other hand, the results of Western blot technique demonstrated that the overexpressing (OE) cell line expressed higher LmAPX compared to wild type (WT) cell line although the complementation (CM) cell lines expressed same amount of LmAPX compared to wild type cells (Fig. 1C–D) [15], [16].

### Increased metacyclogenesis and apoptosis in LmAPX null mutants

**Figure 2:**
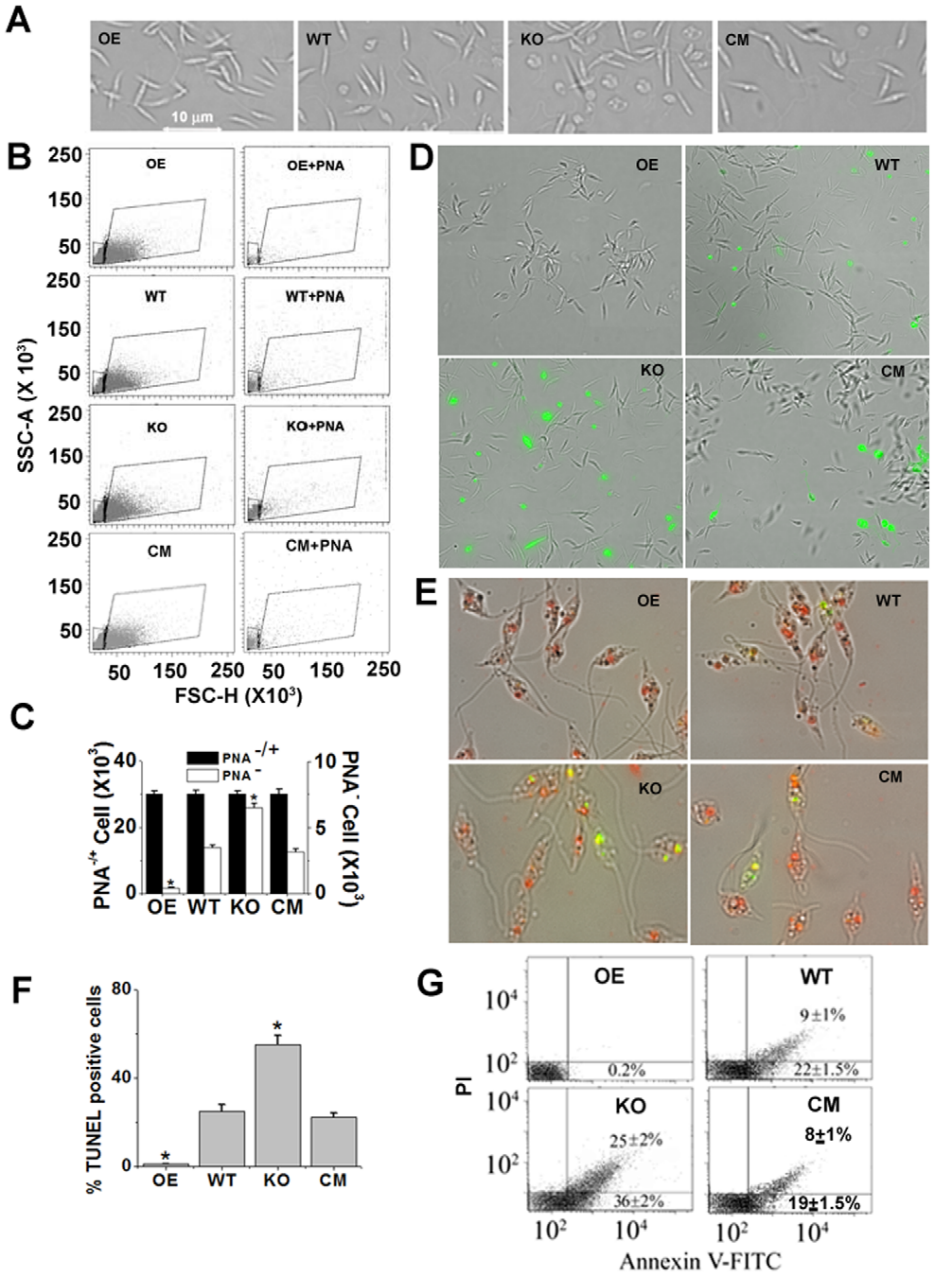
Increased metacyclogenesis and apoptosis in LmAPX null mutants. (A) Bright field images of OE, WT, KO and CM cells. (B) Stationary phase cells were analyzed by flowcytometry before (left panel) and after (right panel) their purification using PNA. Dot plots of FSC-H (forward-angle light scatter) vs. SSC-A (side-angle light scatter) represent the acquisition of 30,000 events. The gating on the dot-plots corresponds to different cell sizes and forward-angle light scatter (FSC) intensities (FSC^low^ and FSC^high^). FSC^low^ (denoted by small box area) represents mainly the metacyclic (PNA^−^) population. (C) Bar diagram of cell number in the total population (PNA^+−^) and PNA^−^ populations of the parasites as derived from (B). Error bars represent the SD from three independent experiments. *Statistically significant value of less than 0.001. (D) AnxA5-FITC staining combined with phase-contrast micrographs are depicted of stationary phase promastigotes. (E) TUNEL-staining of cells counterstained with PI in the presence of RNase. Merged image of cells under bright-field. (F) Bar diagram of % TUNEL positive cells in 300 promastigotes. (G) Cells were double stained with annexin A5 and PI and analyzed by flow cytometric analysis. Dot plots are divided in four quadrants. The lower left, lower right and upper right quadrant represent viable cells, early apoptotic and late apoptotic cells, respectively. Percentages of cells are denoted in the corresponding quadrants. All the data are representative of three independent transfection experiments (three different KO clones).

To investigate whether null mutants (KO) are morphologically distinct from WT, OE or CM cells, microscopic analysis was performed. Microscopic examination showed that KO population had two distinct types of cell compared to WT, OE or CM cells i.e. one type is slender and elongated in shape while the other is round shaped (Fig. 2A). Because typical metacyclic morphology is slender cell body, KO culture might posses the elevated levels of infectious metacyclics. To investigate whether KO culture contained more metacyclics, we had purified metacyclic promastigotes from OE, WT, KO and CM cultures by negative agglutination with peanut agglutinin (PNA). Procyclic promastigotes strongly agglutinate, whereas metacyclic parasites do not react (PNA^−^) due to developmental modifications of terminally exposed oligosaccharides on the lipophosphoglycan [5]. Interestingly, quantitative analysis of PNA^−^ promastigotes by flowcytometry showed that KO cell culture exhibited 16 folds excess metacyclic (PNA^−^) parasites compared to OE cells and similarly the percentage of metacyclic KO (PNA^−^) parasites was 2 folds higher than that of WT or CM cell (Fig. 2B–C). These results strongly suggested that the higher levels of metacyclic parasites were present in the KO population compared to OE, WT or CM cells. As morphology of apoptotic cell is round shaped, knockout culture might present the higher levels of apoptotic cells. We checked whether null mutant populations in liquid culture contain apoptotic (AnxA5+) parasites. Phosphatidylserine exposure is one of the characteristics of apoptotic cells. To study the presence of phosphatidylserine on the surface, *L. major* promastigotes were stained with AnxA5-FITC. Fluorescence microscopy data revealed that a major population of the null mutant parasites was AnxA5^+^ (Fig. 2D), while almost no AnxA5^+^ were found in the OE population. To further substantiate the higher % of apoptotic cell in the null mutant compared with control or overexpressing cells, the DNA fragmentation of AnxA5-analyzed samples was performed in parallel by TUNEL assay (Fig. 2E–F); the results clearly confirmed the data obtained by AnxA5-FITC result in fluorescence microscope. Representative FACS dot plots of FITC-conjugated annexin A5- and PI-labeled samples are shown in Fig. 2G. Quantitative analysis of FITC-conjugated annexin A5- and PI-labeled samples by flowcytometry showed that KO cell culture exhibited 1.6 folds excess early apoptotic (lower right quadrant) and 2.8 folds excess late apoptotic (upper right quadrant) parasites compared to WT or CM cells whereas both early and late apoptotic parasites were absent in OE cell population. These data strongly indicated that the elevated levels of both early and late apoptotic parasites were present in the KO population compared to OE or WT cells.

### Deletion of LmAPX in *L. major* renders the cells sensitive to ROS

**Figure 3:**
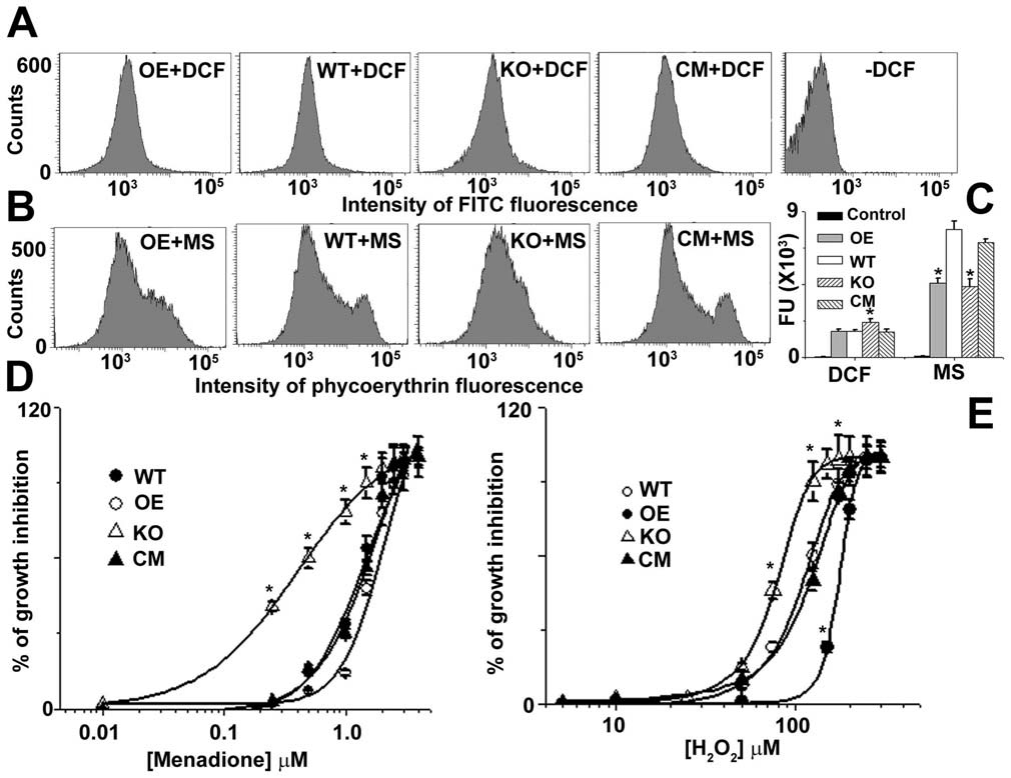
Comparative ROS scavenging study of WT, KO, OE and CM *L. major* cells. (A) Flowcytometric determination of intracellular H_2_O_2_ by DCFDA. (B) Flowcytometric determination of intracellular superoxide by MitoSox Red (MS). (C) Bar diagram of fluorescence intensities depicted in (A) and (B). Relative change in fluorescence in (A) and (B) was analyzed with FACSDiVa software. (D) and (E) are % growth inhibition in presence of various concentration of menadione and H_2_O_2_ respectively. The IC_50_ value for menadione in KO, WT, OE and CM cells was ∼0.36±0.2, 1.38±1.0, 1.8±0.9 and 1.4±0.5 µM respectively. The IC_50_ value for H_2_O_2_ in KO, WT, OE and CM cells was ∼80±5, 110±4.0 and 175±12, 120±10 µM respectively. All data shown are means ± SEM from three independent experiments. *Statistically significant value of less than 0.05. All the data are representative of three independent transfection experiments.

To evaluate the consumption of ROS in OE, WT, KO and CM cell lines, we measured intracellular levels of H_2_O_2_ and O_2_ **^−^** by using the fluorescent probes DCFDA and MitoSox Red, respectively. The intracellular H_2_O_2_ concentration in the KO population was increased compared to the WT, OE or CM cells indicating that LmAPX might have the role in detoxification of endogenous H_2_O_2_ (Fig. 3A–C). Similarly O_2_ **^−^** level in overexpression system was lower than the WT or CM, but it was also lesser in KO (Fig. 3B–C). Because LmAPX-H_2_O_2_ system can oxidize ferrocytochrome c, which scavenges O_2_ **^−^** occasionally, OE cells might eliminate more O_2_ **^−^** compared to WT cells. On the other hand, the low intracellular O_2_ **^−^** concentration in the KO population compared to the WT cells demonstrates that either suppression of O_2_ **^−^** production or elevation of O_2_ **^−^** scavenger has occurred in KO cells. To investigate whether LmAPX lacking cells was sensitive against oxidative stress, the transformed and wild type cell lines were grown in the presence of various concentration of H_2_O_2_ or O_2_ **^−^** generating drug (menadione) and subsequently IC_50_ was determined (Fig. 3D–E). When exposed to exogenous H_2_O_2_ or menadione, the LmAPX overexpressing cell line showed an approximately 1.5 -folds increase in resistance compared with wild type or CM cells. But the KO lines showed a lower IC_50_ value for both H_2_O_2_ and menadione treatment. These results suggested that KO cells were more susceptible to H_2_O_2_ or O_2_ **^−^** compared to LmAPX presenting cells.

### Deletion of LmAPX alleles leads to increased expression of other antioxidant mRNA

**Figure 4:**
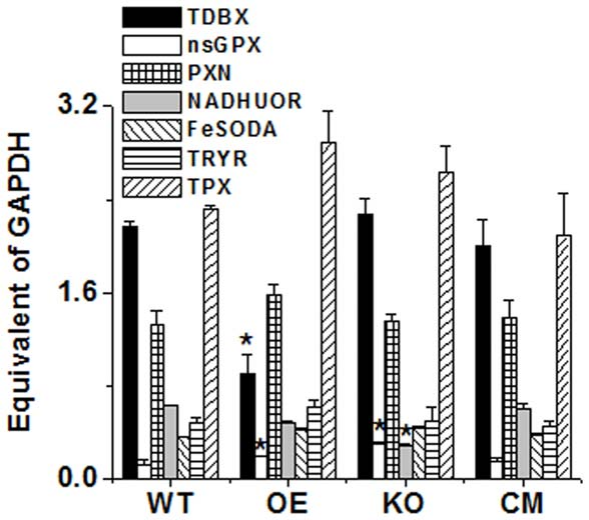
mRNA status of different antioxidant genes in WT, KO, OE and CM in *L. major* cells. Measurements of gene transcript abundance was analyzed by using quantitative RT-PCR as detailed under “Material Methods.” nsGPX, non selenium glutathione peroxidase; TDBX, Type-II tryparedoxin peroxidase; PXN, peroxidoxin tryparedoxin peroxidase; NADHUOR, NADH-ubiquinone oxidoreductase; FeSODA, iron superoxide dismutase; TRYR, trypanothione reductase; TRYP5, tryparedoxin peroxidase. All data were normalized using WT cells glyceraldehyde-3-phosphate dehydrogenase gene (GAPDH, LmjF30.2970) as the endogenous control. Data show mean ± SD of three independent experiments. *P<0.05, compared with WT sample. All the data are representative of three independent transfection experiments.

To investigate the relative quantities of other antioxidant genes in KO, WT, OE and CM cells, we performed quantitative Real time PCR to measure putative non-selenium glutathione peroxidase (nsGPX, LmjF36.3010), Type-II (glutathione peroxidase-like) tryparedoxin peroxidase (TDBX, LmjF26.0810), putative NADH-Ubiquinone oxidoreductase (NADHUOR, LmjF05.0980), iron superoxide dismutase (FESODA, LmjF08.0290), trypanothione reductase (TRYR, LmjF05.0350), tryparedoxin peroxidase (TRYP5, LmjF15.1120) and peroxidoxin tryparedoxin Peroxidase (PXN, LmjF23.0040). qRT-PCR results demonstrated a higher expression of nsGPX (>2 fold) mRNA in KO mutants compared to WT or CM cells (Fig. 4). These results indicated that nsGPX was induced to balance for LmAPX deletion. The expression of NADHUOR was ∼2 fold reduced and that of FeSODA was ∼1.25 fold increased in KO mutants, while the expression levels of the other anti-oxidant genes tested did not differ much (Fig. 4). A possible explanation for lower O_2_^−^ concentration in KO compared to WT (Fig. 3B–C) might be higher FeSODA expression as well as lower level of NADHUOR, which, being part of the respiratory complex I, is a chief site of electron leakage. However, the mRNA expression of TDBX in OE cells was ∼2.5 folds lower compared to WT cells, while nsGPX in OE cells was ∼1.5 folds increased suggesting that peroxide scavenging gene depletion might have induced nsGPX.

### Deletion of LmAPX alleles leads to increased disease development

**Figure 5:**
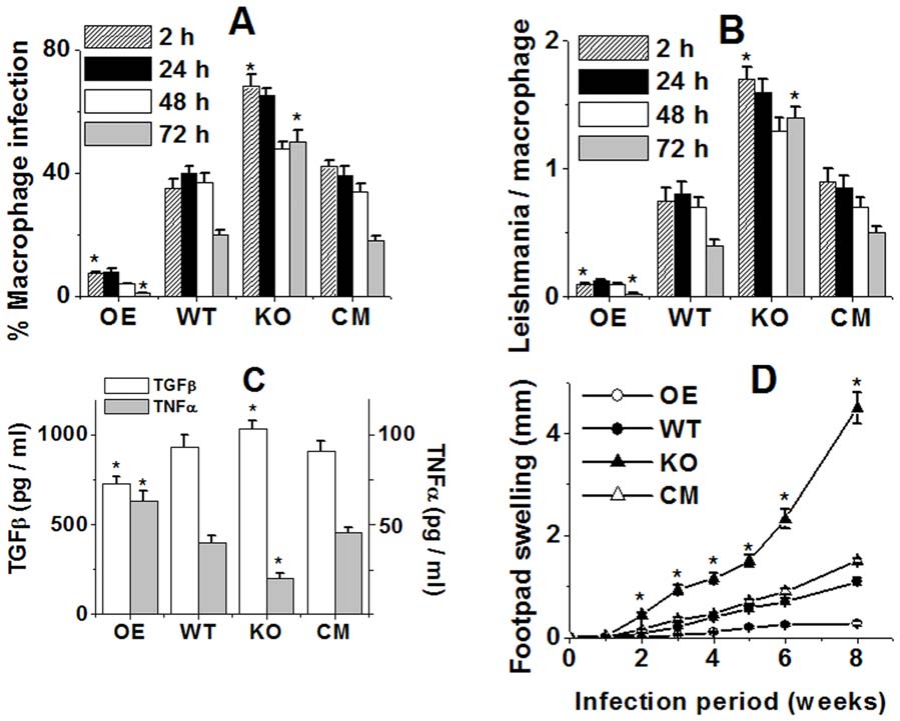
*In vitro* infection of mouse macrophages with WT, KO, OE and CM *L. major* and cytokine production by the phagocytes and footpad lesion development following infection in susceptible BALB/c mice. (A) The percentage of macrophages infected with WT, KO, OE and CM parasites. For each time point 200 macrophages were counted. (B) The number of *Leishmania* within each infected macrophage was counted. For each time point and cell type 100 infected macrophages were analysed. (C) The TNF-α and TGF-β content of the supernatants. Experiments were performed with *n* = 4. Data are given as means ± SD. *P<0.01; compared with control sample. (D) Infection in susceptible BALB/c mice. Data are the mean ± SD of three independent experiments with three groups (8 mice/group) for WT, OE, KO and CM each. *P<0.01. All the data are representative of three independent transfection experiments.

Macrophages are the host cells for *L. major* promastigotes, therefore we focused on promastigote interactions with macrophages. We investigated to what extent KO, WT, OE and CM cells were phagocytosed by macrophages. The internalization rates of KO promastigotes were much higher than WT, CM and OE (Fig. 5A). After phagocytosing KO promastigotes, most of the infected macrophages still contained parasites after 72 h of incubation. However, the percentage of macrophages infected with wild type decreased significantly. In addition, the OE parasites resulted in negligible macrophage infection (Fig. 5B). These data of OE parasites suggested that the parasites were killed more easily inside macrophages in absence of any apoptotic parasite in the inoculum. In general, phagocyte functions are suppressed by the apoptotic cells (AnxA5^+^) via the release of antiinflammatory cytokines like TGF-β and down-regulation of the proinflammatory cytokine TNF-α [7], [20], [21]. The release of TGF-β and TNF-α was measured in supernatants of mouse macrophages after coculture with KO, WT, CM and OE promastigotes. The KO promastigotes induced the release of high levels of TGF-β and down-regulated the expression of the proinflammatory cytokine TNF-α (Fig. 5C) compared to wild type, CM or LmAPX overexpressing cells, probably due to the presence of apoptotic cells, which might be an important factor controlling TGF-β and TNF-α production by monocytes [9], [22], [23]. We later investigated whether *L. major* parasites lacking LmAPX were able to induce infections in mice. After promastigotes were grown to stationary phase, they were inoculated into the footpads of BALB/c mice. We found that KO cells induced a more severe disease with an earlier onset of footpad necrosis, as compared to WT or CM promastigotes (Fig. 5D). These data were confirmed by the OE promastigote infection results, where they were avirulent in an *in vivo* infection model. These findings indicated that the down-regulation of LmAPX gene in parasites is crucial for disease development.

## Discussion

For the single-celled *Leishmania* parasites, which reside in the phagolysosomes of macrophages, the absence of catalase and selenium-containing glutathione peroxidase raises natural curiosity to look for an effective enzyme system capable of detoxification of ROS, and ascorbate peroxidase (LmAPX) from *L. major* presents itself to be a potential candidate. Here, we have successfully generated *L. major* cells completely lacking LmAPX by means of homologous gene replacement method. Our study for the first time depicts a contribution of the leishmanial APX in the infectivity of the parasite to host macrophages and survivability within BALB/c mice. Mutating essential genes, those are implicated in virulence, usually diminishes virulence, pathology, or infectivity. This makes the hypervirulence of the LmAPX null mutants unusual but not unique among *Leishmania* genes. The pteridine reductase 1 null mutants in *Leishmania* results in hypervirulence manifested by increased lesion formation and parasite numbers, probably due to the action of tetrahydrobiopterin, which may be an important factor controlling metacyclogenesis [24]. Although our result shows LmAPX levels as another important factor for controlling the extent of both metacyclogenesis and apoptosis but the mechanism is completely different from tetrahydrobiopterin depletion mechanism. The apoptotic *Leishmania* in KO culture exhibits altruistic behavior in providing survival advantage for the viable metacyclic parasites.

At least three central players are involved in the removal of H_2_O_2_ in *Leishmania*: 2-cysteine peroxiredoxins, nsGPX, and LmAPX [25]. From our recent study it is revealed that as the culture is incubated with H_2_O_2_, where the cells encounter more oxidative stress, the expression of the LmAPX enzyme also increases [16]. Therefore, it is clear that in the absence of catalase, the single copy LmAPX gene plays a major role in protecting the parasite against oxidative stress. In this study we reported that a deficiency in LmAPX resulted in about two-fold induction of nsGPX, along with a slight induction of peroxidoxin and type II (glutathione peroxidase-like) tryparedoxin peroxidase, suggesting that these enzymes were induced to compensate for LmAPX. Taken together, these findings suggest a high degree of redundancy in ROS detoxifying mechanisms in *Leishmania*. Interestingly, compared to OE, CM or WT *Leishmania*, KO cells have been more sensitive to ROS stress. This is in accordance with the previous finding which points to the low K_m_ value of LmAPX for H_2_O_2_ (∼25 µM in presence of ascorbate).

However, at least within macrophage, KO *Leishmania* did not appear to be subjected to the same degree of oxidative stress as OE, CM or WT *Leishmania*. It is possible that KO *Leishmania* induced an unknown ROS scavenging mechanism that protected them from ROS-induced damage. Alternatively, they might have suppressed the rate of ROS production within phagolysosome, thereby lowering the intracellular level of ROS. The suppression of pro-inflammatory activity or induction of antiinflammatory activity in macrophage by KO *Leishmania* may account for such a response. The oxidative burst is one of the major sources of ROS production within macrophage, and suppression of oxidative burst was shown to accompany the defense response of certain *Leishmania* to adverse physical conditions [26], [27].

It has been established from our previous studies that LmAPX plays an important role for scavenging of ROS [16]. Accordingly, here null mutant shows elevated level of intracellular H_2_O_2_ as well as lower level of IC_50_ value for both exogenously added H_2_O_2_ and superoxide generating drug (menadione). Thus, the hypervirulence observed for LmAPX null mutants occurs through a distinct mechanism. A central question in the study of *Leishmania* is why KO cells cause cutaneous leishmaniasis at the site of infection, whereas OE parasites produce little or no cutaneous pathology. To survive the hostile environment of macrophages, *Leishmania* developed subversion mechanisms including the modulation of host cytokine production. Pro-inflammatory cytokine TNF-α as well as anti-inflammatory cytokine TGF-β can tightly control the intracellular replication of both *Leishmania* and *T. cruzi in vitro* and *in vivo* by modulating the antimicrobial activities of the phagocytes [28], [29]. Here, TNF-α levels were significantly higher for OE- compared to WT-, CM- or KO-infected macrophages. On the other hand, TGF-β levels were significantly lower for OE- compared to WT-, CM- or KO-infected macrophages. We cannot exclude that differences in TNF-α or TGF-β production could be responsible for different rates of infection observed between OE and KO strains. In human, some studies demonstrated that *Leishmania* are able to infect monocytes without inducing TNF-α production [30], whereas others report the opposite [31]. TGF-β is well-known in silencing cytotoxic effector reactions of activated macrophage and a direct correlation between the amounts of TGF-β produced following *in vitro* infection of murine macrophages and virulence of *Leishmania* was demonstrated [22], [23]. In agreement with previous studies, we observed both TGF-β and TNF-α modulation by KO during the early inflammatory events which might be associated directly with the outcome of disease.

It is also possible that the loss of APX activity has secondary effects on leishmanial gene expression caused by an alteration in the redox balance in the protozoa. As a consequence, modulation in the form of LPG and simultaneous differentiation of the promastigotes into infective metacyclic form may occur. A group of workers have suggested that the infectious parasite population in the midgut of infected sandfly or *in vitro* stationary phase cultures contain both metacyclic as well as apoptotic parasites and these cells are needed for infectivity of the parasite within host macrophages [9]. In accordance with this, we also found that KO *Leishmania*, deprived of LmAPX, contains in its stationary phase culture a higher number of metacyclic as well as apoptotic parasites compared to both wild type and LmAPX overexpressing cells. Thus, our results show that LmAPX may have a major role in cellular differentiation in *L. major* as well as may act to protect the cells from apoptosis. There is now a wealth of evidence to suggest that H_2_O_2_ could act directly as a signaling molecule, by affecting cell differentiation, signal transduction and cell death in other organisms [12]. Hence, besides protection against apoptosis we have identified a new and second role for LmAPX in *Leishmania*, that of regulating parasite differentiation to the infective metacyclic stage by modulating the ROS content of the cell.

## Materials and Methods

### Reagents

2', 7'-dichlorodihydrofluorescein diacetate (DCFDA), MitoSOX™ Red were procured from Molecular Probes (Eugene, OR). Anti α-tubulin antibody was purchased from Upstate Cell Signaling. All other chemicals were purchased from Sigma (St Louis.MO) or sources previously reported [16], [17], [32]–[35].

### Ethics Statement

All Balb/C mice were obtained from and maintained in our Institutional animal facility (Kolkata, India). The studies were approved by IICB Animal Ethical Committee (Registration no. 147/1999, CPCSEA), registered with Committee for the purpose of Control and Supervision on Experiments on Animals (CPCSEA), Govt. of India, and Balb/C mice were handled according to their guidelines.

### Molecular constructs for overexpression of LmAPX allele

*L. major* (strain 5ASKH) promastigotes were cultured at 26°C in M199 medium supplemented with 40 mM HEPES, (pH-7.4) 200 µM adenine, 1% penicillin-streptomycin (v/v), 50 µg/ml gentamycin and 10% heat inactivated fetal bovine serum [16], [17]. Genomic DNA was isolated from *L. major* promastigotes by Qiagen genomic DNA isolation kit as per manufacturer's instructions. To amplify LmAPX open reading frame, following primers were used. Primer1: 5′-AAAACCCGGGCGCATGTCCGGCACCTCGCGG-3′, Primer2: 5′-AAAAGGATCCTTAGCTCTCCGAAGCGGGTGCTTTG-3′. The underlined portions denote restriction enzyme sites. The amplified product of primer1 and primer2 was cloned in pXG-B2863 vector at SmaI and BamHI sites to generate full length LmAPX. Transformation of the LmAPX containing pXG-B2863 vector in *Leishmania* cell was performed by electroporation with a Bio-Rad Gene Pulsar apparatus using 450 volt and 550 µF capacitance [16]. Finally all the transfected cells were maintained in 200 µg/ml G418.

### Modification of the vector and molecular constructs for knockout of LmAPX alleles

The vectors pXG-neo and pXG-hyg were first modified by site-directed mutagenesis at position 1981 and 1952 respectively at the T3 promoter end of the vectors to generate unique HindIII site using following primers: Primer3: 5′-CACACACAAAGCTGCCTTGCACACAACG-3′ and Primer4: 5′-CGTTGTGTGCAAGGCAGCTTTGTGTGTG-3′. The underlined portions denote the site of mutation. Next, a 0.976 Kb flanking sequence upstream (5′F) and 1.051 Kb flanking sequence downstream (3′F) of LmAPX were PCR amplified from *L. major* genomic DNA using the following primers: Primer5: 5′-AAAAGGATCCCCCTCCTCTGTCAAGTGTG-3′, Primer6: 5′-AAAAGAATTCGCGTGGCACGTAACTAGGC-3′, Primer7: 5′-AAAACCCGGGGACCGAGCTCGGCACCAGG-3′, and Primer8: 5′-AAAAGGATCCCACCTGACCCACTGCACAGC-3′. The primers 5 and 6 were used to amplify the 5′F and the primers 7 and 8 were used to amplify the 3′F. The 5′F was then cloned at the *BamHI/EcoRI* site of the pTrcHisA vector and an internal HindIII site at position 307 of 5′F was modified by site-directed mutagenesis using primers: Primer9: 5′-CACTCGTATCGGCGAGCTGCGCTGTACACTGC-3′ and Primer10: 5'- GCAGTGTACAGCGCAGCTCGCCGATACGAGTG-3′. The modified 5′F (mod5′F) was next PCR amplified from pTrcHisA with the help of the primers: Primer11: 5′-AAAAAAGCTTCCCTCCTCTGTCAAGTGTG-3′ and Primer6. Next, the mod5′F was digested with HindIII and SalI restriction enzymes and cloned at the HindIII/SalI site of the vectors pXG-neo and pXG-hyg. Then, the 3′F was cloned in the vectors containing mod5′F at SmaI/BamHI site. These were then digested with HindIII and BamHI to generate the gene deletion constructs LmAPX::NEO and LmAPX::HYG.

### Generation of LmAPX knockout cells

For generating LmAPX knockout *L. major*, transformations of all the constructs were performed by electroporation [16], [36] with a Bio-Rad Gene Pulsar apparatus using 450 volt and 550 µF capacitance. Briefly late log phase wild type (WT) promastigotes (0.5-1.0×10^7^) were harvested at 1200 g (4°C) for 10 minutes and washed twice in electroporatic buffer (21 mM HEPES, 137 mM NaCl, 0.7 mM NaH_2_PO_4_, 6 mM glucose, pH 7.4). Cells were finally suspended in a density of 1×10^8^/ml and 0.36 ml was taken in a 0.2 mm ice chilled electroporation cuvette. 12 µg of linear gene replacement cassette LmAPX::NEO, dissolved in 40 µl of electroporatic buffer, was then added to cuvette and incubated in ice for 10 minutes followed by electroporation. Cells were incubated further for 10 minutes in ice and added to 10 ml of drug free growth medium. After 24 hours of revival, 20 µg/ml G418 added and kept at 26°C for another 10 days with mild shaking. Finally all the transfected cells (LmAPX^−/+^ mutants) were maintained in 50 µg/ml G418. The latter was selected for the second round of targeting using the LmAPX::HYG construct. Transfectants (LmAPX^−/−^ or KO) resistant to both G418 and Hygromycin B were obtained and clones were maintained in 50 µg/ml G418 and 100 µg/ml hygromycin B.

### Complementation of LmAPX in null mutants

To restore LmAPX in the knock out parasites, LmAPX orf was first PCR-amplified using a LmAPX containing plasmid as template and the following forward and reverse primers. Forward primer: 5′-AAAAGGATCCCGCATGTCCGGCACCTCGCGG-3′. Reverse primer: 5′-AAAAGGATCCTTAGCTCTCCGAAGCGGGTGCTTTG-3′. The underlined portions denote BamHI restriction enzyme sites. The amplified product was cloned at the same site of pXG-PHLEO vector and 10 µg of the recombinant plasmid, pXG-PHLEO-LmAPX, was transfected into the knockout promastigotes. Transfected promastigotes were selected with minimal doses of phleomycin (5 µg/ml) and finally grown in the presence of 10 µg/ml of drug. A series of transfected clones were picked up for measuring LmAPX expression by Western blot analysis with anti-LmAPX antibody. We used CM clones, which were expressed equal amount of LmAPX compare to wild type cells.

### Microscopic Analysis

After fixation with paraformaldehyde, WT, OE KO and CM cells from late log phase were observed under the Olympus IX81microscope for bright field imaging. At least 20 microscope fields were observed for each sample.

### Western blot

Proteins from whole cell were resolved on 13% SDS-PAGE and electroblotted onto nitrocellulose membranes (Sigma) by Hoefer wet transfer apparatus. Membranes were incubated with rabbit anti LmAPX (1∶50) polyclonal antibody followed by anti rabbit (1∶5000) secondary antibody. Detection was carried out by enhanced chemiluminescence reaction by ECL kit (Amersham). Expression of α-tubulin was considered as the endogenous control.

### Real-time quantitative PCR

Real time PCR was performed to investigate the relative quantities of antioxidant genes like Glutathione Peroxidase (nsGPX), type-II (Glutathione Peroxidase-like) tryparedoxin peroxidase (TDBX), NADH-ubiquinone oxidoreductase (NADHUOR), iron superoxide dismutase (FESODA), trypanothione reductase (TRYR), tryparedoxin peroxidase (TRYP5) and peroxidoxin tryparedoxin Peroxidase (PXN) in WT, OE, KO and CM cells. Briefly, total RNA was isolated from the parasites using RNAqueous®-4PCR Kit (Ambion) according to the manufacture's protocol. cDNA synthesis was then performed using High Capacity cDNA Reverse Transcription Kit (Applied Biosystems, ABI). Real-time quantitative PCR was performed on the StepOne Real-Time PCR system (Applied Biosystems, ABI) using *Power* SYBR® Green PCR Master Mix (ABI) and primers (Table 1). Relative expression levels of mRNA were normalized using WT cells as reference sample and Glyceraldehyde-3-phosphate dehydrogenase gene (GAPDH, LmjF30.2970) as the endogenous control using a Comparative C_T_ method as described by the manufacturer.

**Table 1:** To amplify the relative quantities of antioxidant genes, following primers were used.

Gene	Forward Primer	Reverse Primer
TDBX	5′-CAGCGACCACCAGCCATAC-3′	5′-TCACCTCCTCCTCCGTTCC-3′
nsGPX	5′-TGGCATCCGTCTTCACCT-3′	5′-CACCTCGTTCAGCATCTCG-3′
PXN	5′-TCGCTCGTGACTACGGTGTG-3′	5′-GCTGCCTTTGTGGTGTCCAG-3′
NADHUOR	5′-GGTGAGGAGACGGCGATGA-3′	5′-GGCGGATTATGGTCGGTGAC-3′
FESODA	5′-TCGGGTTTTCGTGCCTGT-3′	5′-TCGGGTTTTCGTGCCTGT-3′
TRYR	5′-GCACGGAGGAGGACTACGA-3′	5′-ATCGGCGGTATGGAGAACAC-3′
TRYP5	5′-GTGGGTCGTGCTCTTCTTCTAC-3′	5′-GCTCTTGGTCTTGTCGGCTA-3′
GAPDH	5′-TCAAGGGTGGTGCGAAGAAG-3′	5′-TCGCCGTGTAGGAGTGGATG-3′

### Determination of IC_50_

Growth inhibitory concentration of H_2_O_2_ or Menadione was studied on WT, OE, KO and CM cells using Nunclon 24 well plates. Log phase promastigotes of WT, OE KO and CM cells were seeded at 3×10^5^ cells/ml in 2 ml growth medium in presence of increasing concentration of H_2_O_2_ or Menadione. After five days of growth, cell densities were determined by haemocytometer.

### Flowcytometric measurement of intracellular ROS

To detect ROS, cells were incubated with O_2_^−·^-sensitive probe MitoSOX™ Red (Ex/Em: 488/580) and H_2_O_2_-sensitive probe DCFDA (Ex/Em: 488/530). Briefly, 5×10^6^ cells were collected by centrifugation, washed, and then resuspended in PBS containing 5 µM MitoSOX™ Red or 6 µM DCFDA for 25 minutes at room temperature. Cells were further washed twice in PBS and fluorescence was measured subsequently by FacsCanto flowcytometer (Becton Dickinson, San Jose, CA). Relative change in fluorescence was analyzed with FACSDiVa software.

### Apoptosis assessment by Annexin-A5 staining

Phosphatidyl serine (PS) exposure was assessed by Vybrant apoptosis assay kit # 3 (Molecular Probes). WT, OE, KO and CM cells were harvested by centrifugation for 5 min at 1200 g and washed twice with cold PBS. Cells (1×10^6^/ml) were then resuspended in 100 µl 1X annexin binding buffer and incubated with 5 µl FITC conjugated annexin A5 and 1 µg/ml PI for 15 min. After staining, 400 ml of 1X buffer was added to the cells, and samples were stored on ice until data acquisition by flow cytometry. To eliminate the emission spectral overlap of fluophores, fluorescence compensation was performed with log phase unstained and single-stained (with either PI or with FITC) promastigotes. For fluorescence imaging, only annexin A5-bound cells were used.

### TUNEL staining

Cells undergoing apoptosis go through DNA fragmentation in the nuclei. TUNEL staining was performed with an Apoalert DNA fragmentation assay kit (Clontech, Mountain View, CA) to detect in *vivo* DNA fragmentation according to manufacturer's manual as described before [15].

### Metacyclic purification assay and flowcytometry

Metacyclic promastigotes were purified from stationary cultures by treatment with the PNA lectin as described before [37]. Briefly, stationary promastigote cultures of WT, OE, KO and CM parasites were washed in PBS, resuspended to 10^8^/ml cell density, and incubated with 50 µg/ml of PNA. After 30 min of incubation, the suspension was centrifuged at 40 *g* for 5 min. The non-agglutinated promastigotes (PNA^−^), collected in the supernatant, were washed two times in PBS and resuspended in PBS and analyzed for light scatter by Flowcytometry. Dot plots of forward-angle light scatter (FSC) vs. side-angle light scatter (SSC) represent the acquisition of 30,000 events [38].

### In vitro macrophage infection

Promastigotes were used to infect cultures of adherent murine macrophage cell line RAW 264.7 on glass coverslips (22 mm^2^; 5×10^5^ macrophages/coverslip) in 0.5 ml of RPMI 1640/10% FCS at a parasite to cell ratio of 10∶1 for a period of 2 hrs for determination of parasite entry and a period of 6 hrs for determination of intracellular parasite numbers. Following the incubation, unphagocytosed parasites were removed by washing with medium, and cells were resuspended in RPMI 1640/10% FCS at 37°C, 5% CO_2_ and then 6 hrs incubated cultures were transferred to a CO_2_ incubator at 37°C for an infection period of 24 hrs, 48 hrs and 72 hrs in presence of RNase A. Cells were then fixed in methanol and stained with propidium iodide. Cells were visualized and quantified using Olympus IX81 microscope.

### Cytokine measurements

TGF-β and TNF-α production by macrophages was assessed by ELISA (OptEIA™ TGF-β1 ELISA Set and OptEIA™ TNF (Mono/Mono) ELISA Set (BD Biosciences) respectively according to manufacturer's protocol.

### Infection in mice

For cutaneous infection, female BALB/c mice, 6–7 weeks old, were infected subcutaneously with 5×10^6^ stationary-phase WT, OE, KO and CM promastigotes in their left hind footpads [8 mice/group). Disease progression was monitored by daily caliper measurement of footpad swelling.

### Statistical analysis

All results were expressed as the mean ± SE from at least three independent experiments. Statistical analysis for parametric data was calculated by Student's *t* test or analysis of variance (ANOVA) wherever applicable using Origin 7.0 software (Microcal software, Inc. Northampton, MA, USA). The ANOVA was followed by post hoc analysis (multiple comparison *t* test) for the evaluation of the difference between individual groups. A *p* value of less than 0.05 was considered statistically significant.

## References

[1] Sacks DL, Perkins PV (1985). Development of infective stage *Leishmania* promastigotes within phlebotomine sand flies.. Am J Trop Med Hyg.

[2] Sacks DL (1989). Metacyclogenesis in *Leishmania* promastigotes.. Exp Parasitol.

[3] McConville MJ, Turco SJ, Ferguson MA, Sacks DL (1992). Developmental modification of lipophosphoglycan during the differentiation of *Leishmania major* promastigotes to an infectious stage.. Embo J.

[4] Sacks DL, Brodin TN, Turco SJ (1990). Developmental modification of the lipophosphoglycan from *Leishmania major* promastigotes during metacyclogenesis.. Mol Biochem Parasitol.

[5] Sacks DL, Perkins PV (1984). Identification of an infective stage of *Leishmania* promastigotes.. Science.

[6] da Silva R, Sacks DL (1987). Metacyclogenesis is a major determinant of *Leishmania* promastigote virulence and attenuation.. Infect Immun.

[7] Voll RE, Herrmann M, Roth EA, Stach C, Kalden JR (1997). Immunosuppressive effects of apoptotic cells.. Nature.

[8] Henson PM (2004). Fingering IL-12 with apoptotic cells.. Immunity.

[9] van Zandbergen G, Bollinger A, Wenzel A, Kamhawi S, Voll R (2006). *Leishmania* disease development depends on the presence of apoptotic promastigotes in the virulent inoculum.. Proc Natl Acad Sci U S A.

[10] Wanderley JL, Pinto da Silva LH, Deolindo P, Soong L, Borges VM (2009). Cooperation between apoptotic and viable metacyclics enhances the pathogenesis of Leishmaniasis.. PLoS One.

[11] Rhee SG (2006). Cell signaling. H_2_O_2_, a necessary evil for cell signaling.. Science.

[12] Sundaresan M, Yu ZX, Ferrans VJ, Irani K, Finkel T (1995). Requirement for generation of H_2_O_2_ for platelet-derived growth factor signal transduction.. Science.

[13] Cadenas E (2004). Mitochondrial free radical production and cell signaling.. Mol Aspects Med.

[14] Choi WS, Eom DS, Han BS, Kim WK, Han BH (2004). Phosphorylation of p38 MAPK induced by oxidative stress is linked to activation of both caspase-8- and -9-mediated apoptotic pathways in dopaminergic neurons.. J Biol Chem.

[15] Dolai S, Yadav RK, Pal S, Adak S (2009). Overexpression of Mitochondrial *Leishmania major* ascorbate peroxidase shows enhanced tolerance to oxidative stress-induced programmed cell death and protein damage.. Eukaryot Cell.

[16] Dolai S, Yadav RK, Pal S, Adak S (2008). *Leishmania major* ascorbate peroxidase overexpression protects cells against reactive oxygen species-mediated cardiolipin oxidation.. Free Radic Biol Med.

[17] Adak S, Datta AK (2005). *Leishmania major* encodes an unusual peroxidase that is a close homologue of plant ascorbate peroxidase: a novel role of the transmembrane domain.. Biochem J.

[18] Flohe L, Ursini F (2008). Peroxidase: a term of many meanings.. Antioxid Redox Signal.

[19] Cruz A, Coburn CM, Beverley SM (1991). Double targeted gene replacement for creating null mutants.. Proc Natl Acad Sci U S A.

[20] Fadok VA, McDonald PP, Bratton DL, Henson PM (1998). Regulation of macrophage cytokine production by phagocytosis of apoptotic and post-apoptotic cells.. Biochem Soc Trans.

[21] Fadok VA, Bratton DL, Konowal A, Freed PW, Westcott JY (1998). Macrophages that have ingested apoptotic cells in vitro inhibit proinflammatory cytokine production through autocrine/paracrine mechanisms involving TGF-beta, PGE2, and PAF.. J Clin Invest.

[22] Barral A, Barral-Netto M, Yong EC, BrownellCE, Twardzik DR (1993). Transforming growth factor beta as a virulence mechanism for *Leishmania braziliensis*.. Proc Natl Acad Sci U S A.

[23] Barral A, Teixeira M, Reis P, Vinhas V, Costa J (1995). Transforming growth factor-beta in human cutaneous leishmaniasis.. Am J Pathol.

[24] Cunningham ML, Titus RG, Turco SJ, Beverley SM (2001). Regulation of differentiation to the infective stage of the protozoan parasite *Leishmania major* by tetrahydrobiopterin.. Science.

[25] Castro H, Tomas AM (2008). Peroxidases of trypanosomatids.. Antioxid Redox Signal.

[26] Murray HW, Nathan CF (1988). In vivo killing of intracellular visceral Leishmania donovani by a macrophage-targeted hydrogen peroxide-generating system.. J Infect Dis.

[27] Murray HW, Nathan CF (1999). Macrophage microbicidal mechanisms in vivo: reactive nitrogen versus oxygen intermediates in the killing of intracellular visceral Leishmania donovani.. J Exp Med.

[28] Nelson BJ, Ralph P, Green SJ, Nacy CA (1991). Differential susceptibility of activated macrophage cytotoxic effector reactions to the suppressive effects of transforming growth factor-beta 1.. J Immunol.

[29] Silva JS, Twardzik DR, Reed SG (1991). Regulation of Trypanosoma cruzi infections in vitro and in vivo by transforming growth factor beta(TGF-beta).. J Exp Med.

[30] Reiner NE, Ng W, Wilson CB, McMaster WR, Burchett SK (1990). Modulation of in vitro monocyte cytokine responses to Leishmania donovani. Interferon-gamma prevents parasite-induced inhibition of interleukin 1 production and primes monocytes to respond to Leishmania by producing both tumor necrosis factor-alpha and interleukin 1.. J Clin Invest.

[31] Green SJ, Crawford RM, Hockmeyer JT, Meltzer MS, Nacy CA (1990). Leishmania major amastigotes initiate the L-arginine-dependent killing mechanism in IFN-gamma-stimulated macrophages by induction of tumor necrosis factor-alpha.. J Immunol.

[32] Adak S, Bilwes AM, Panda K, Hosfield D, Aulak KS (2002). Cloning, expression, and characterization of a nitric oxide synthase protein from Deinococcus radiodurans.. Proc Natl Acad Sci U S A.

[33] Dolai S, Yadav RK, Datta AK, Adak S (2007). Effect of thiocyanate on the peroxidase and pseudocatalase activities of Leishmania major ascorbate peroxidase.. Biochim Biophys Acta.

[34] Yadav RK, Dolai S, Pal S, Adak S (2008). Role of tryptophan-208 residue in cytochrome c oxidation by ascorbate peroxidase from Leishmania major-kinetic studies on Trp208Phe mutant and wild type enzyme.. Biochim Biophys Acta.

[35] Adak S, Aulak KS, Stuehr DJ (2002). Direct evidence for nitric oxide production by a nitric-oxide synthase-like protein from Bacillus subtilis.. J Biol Chem.

[36] Kapler GM, Coburn CM, Beverley SM (1990). Stable transfection of the human parasite Leishmania major delineates a 30-kilobase region sufficient for extrachromosomal replication and expression.. Mol Cell Biol.

[37] Sacks DL, Hieny S, Sher A (1985). Identification of cell surface carbohydrate and antigenic changes between noninfective and infective developmental stages of *Leishmania major* promastigotes.. J Immunol.

[38] Saraiva EM, Pinto-da-Silva LH, Wanderley JL, Bonomo AC, Barcinski MA (2005). Flow cytometric assessment of *Leishmania* spp metacyclic differentiation: validation by morphological features and specific markers.. Exp Parasitol.

[39] Gurunathan S, Sacks DL, Brown DR, Reiner SL, Charest H (1997). Vaccination with DNA encoding the immunodominant LACK parasite antigen confers protective immunity to mice infected with Leishmania major.. J Exp Med.

